# Sex-specific difference in phenotype of Kabuki syndrome type 2 patients: a matched case-control study

**DOI:** 10.1186/s12887-024-04562-z

**Published:** 2024-02-19

**Authors:** Yirou Wang, Yufei Xu, Yao Chen, Yabin Hu, Qun Li, Shijian Liu, Jian Wang, Xiumin Wang

**Affiliations:** 1 Department of Endocrinology and Metabolism, Shanghai Children’s Medical Center,, School of Medicine, Shanghai Jiao Tong University, Shanghai, China; 2 Department of NeurologySchool of Medicine, Shanghai Children’s Medical Center, School of Medicine, Shanghai Jiao Tong University, Shanghai, China; 3 Children Health Advocacy Institute, Shanghai Children’s Medical Center, School of Medicine, Shanghai Jiao Tong University, Shanghai, China; 4 School of Public Health, Shanghai Children’s Medical Center, School of Medicine, Shanghai Jiao Tong University, Shanghai, China

**Keywords:** Kabuki syndrome type 2, *KDM6A*, Sex-specific difference, Severe intellectual disability

## Abstract

**Background:**

Kabuki syndrome (KS) is a monogenic disorder leading to special facial features, mental retardation, and multiple system malformations. Lysine demethylase 6A, (*KDM6A*, MIM*300128) is the pathogenic gene of Kabuki syndrome type 2 (KS2, MIM#300867), which accounts for only 5%–8% of KS. Previous studies suggested that female patients with KS2 may have a milder phenotype.

**Method:**

We summarized the phenotype and genotype of KS2 patients who were diagnosed in Shanghai Children’s Medical Center since July 2017 and conducted a 1:3 matched case–control study according to age and sex to investigate sex-specific differences between patients with and without KS2.

**Results:**

There were 12 KS2 cases in this study, and 8 of them matched with 24 controls. The intelligence quotient (IQ) score of the case group was significantly lower than that of the control group (*P* < 0.001). In addition, both the incidence of intellectual disability (ID) (IQ < 70) and moderate-to-severe ID (IQ < 55) were significantly higher in the case group than those in the control group. No sex-specific difference was found in the incidence of ID or moderate-to-severe ID between the female cases and female controls, whereas there was a significant difference between male cases and male controls. Furthermore, the rate of moderate-to-severe ID and congenital heart disease (CHD) was significantly higher in the male group than that in the female group.

**Conclusions:**

Our results showed that a sex-specific difference was exhibited in the clinical phenotypes of KS2 patients. The incidence of CHD was higher in male patients, and mental retardation was significantly impaired. However, the female patients’ phenotype was mild.

## Background

Kabuki Syndrome (KS) is a rare genetic disease often caused by two pathogenic genes, *KMT2D* (MIM*602113) and *KDM6A* (MIM*300128), with coding for histone methylase and histone demethylase, respectively [1, 2]. *KMT2D* causes KS type 1 (KS1, MIM# 147920), whereas *KDM6A* causes KS type 2 (KS2, MIM# 300867). KS2 only accounts for approximately 5%–8% of KS cases [3–5]. Most patients with KS are characterized by mental retardation, which manifests as infantile hypotonia in infancy, and mild-to-moderate intellectual disability (ID) in childhood. *KDM6A* is located at Xp11.3, and KS2 is X-linked dominant inheritance.

Previous studies have found that the phenotype of KS2 is different from that of KS1, for example, patients with KS1 type have a higher risk of typical facial features, short stature, and frequent infections compared to those with KS2 [6, 7]. Many reports have found that the incidence of ID in KS1 is also higher than that in KS2, hence, researchers proposed that this phenomenon may be caused by sex-specific phenotypic differences in patients with KS2 since female patients with KS2 often show mild clinical manifestations [3, 8]. However, there are few reports of KS2, and, as of April 2020, only 94 variants of *KDM6A* have been included in the Human Gene Mutation Database (http://www.hgmd.cf.ac.uk/ac/index.php). Therefore, the clinical manifestations of patients with KS2 need to be require further summarized and analyzed.

In the present study, we summarized and analyzed 12 KS2 patients with definite *KDM6A* pathogenic variants, including three pedigrees, wherein the pathogenic variant of the proband was inherited from the mother. Additionally, we used a matched case–control study design to evaluate the difference in the incidence of various clinical characteristics of KS2 between the sexes. We hypothesized that KS2 female patients might have a milder phenotype.

## Materials and methods

### Participants and sampling

We enrolled KS patients diagnosed at SCMC between June 2017 and June 2021, and statistically analyzed their clinical phenotypes. To be eligible as a case, genetic testing indicating a *KDM6A* “Pathogenic” or “Likely Pathogenic” variant according to the ACMG guidelines was required [9]. To further explore the gender differences in intelligence levels among KS2 patients, we conducted a case–control study with the age- and gender-matched children as the control group. As for the controls, we selected 200 children who underwent a physical examination and an intelligence test at the Children’s Health Department of SCMC from April 2021 to June 2021. Children diagnosed with organic brain disease (e.g., traumatic brain injury, hydrocephalus, brain tumor, etc.), a history of brain surgery, inherited metabolic diseases (e.g., mitochondrial encephalomyopathy, phenylketonuria, Turner syndrome, and so on), hearing loss, and severe visual impairment that could cause mental retardation were excluded from the study. The KS2 cases and controls were matched at a 1:3 ratio by age and sex.

### Evaluation and diagnosis

The phenotypic assessment of KS was performed by professional genetic clinicians, and evaluation of each patient was based on the clinical symptoms listed in the 2019 international consensus [7]. Molecular testing for KS was performed through whole-genome sequencing, and candidate variants were validated by Sanger sequencing using specific primers. The DNA of the patients’ parents was also isolated and subjected to Sanger sequencing to confirm the origin of the candidate variants. The variants were categorized according to the method recommended by the American College of Medical Genetics and Genomics [9]. Patients with characteristic clinical symptoms and a molecular diagnosis were finally diagnosed with KS2.

### Data collection

Participants’ IQ scores were measured using the Wechsler Preschool and Primary Scale of Intelligence, Wechsler Intelligence Scale for Children–Fifth Edition, and Wechsler Adult Intelligence Scale–Fourth Edition. The severity of ID was categorized as mild (IQ score, 69–55), moderate (IQ score, 54–40), or severe (IQ score, < 40). The calculation of the standard deviation (SD) score for height refers to the growth chart standards for Chinese children and adolescents [10]. Other assessments included a blood cell analysis, routine evaluation of biochemical indicators, immune function analysis, and various imaging examinations (including cardiac ultrasonography, renal ultrasonography, electrocardiography, etc.).

### Statistical analysis

The detection rates of ID, typical dysmorphic features, short stature, microcephaly, etc. were directly calculated, and Pearson’s chi-square test was used to compare rates between the case and control groups. Categorical variables were analyzed using Fisher’s exact test if the expected frequency was less than five. The rank sum test was used for two-group comparisons of age, height, SD scores, and IQ score data. All the raw data were analyzed using SPSS Statistical package version 21 (IBM Corp., Armonk, New York, USA), and a two-sided *P*-value < 0.05 was considered statistically significant.

## Results

### Participants

Only nine patients with KS2 were diagnosed at Shanghai Children’s Medical Center (SCMC) and enrolled in the case group. Interestingly, when we verified the genotype of their parents, we discovered that the variants of three patients were inherited from their mothers. After further evaluation, we found that their mothers could also be diagnosed with KS2. In total, 12 patients formed the case group, five males and seven females; the median age of the males and females was 5.88 ± 4.36 years and 17.98 ± 13.62 years, respectively. There was no statistical difference in age distribution between the female and male patient in case group.

### Phenotype and genotype

We summarized the phenotype of 12 patients with KS2 (Table 1). Most had dysmorphic features typical of KS (11/12, 91.6%), and the most frequent clinical features (frequency ≥ 50%) included feeding difficulties (77.8%), ID (58.3%), and congenital heart defect (CHD) (50.0%). However, when we compared the incidence of common clinical manifestations between the sexes, we found that the rates of moderate-to-severe ID and CHD were significantly higher in the male group than in the female group (both, *P* < 0.05) (Table 2).

**Table 1:** Phenotypic features of 12 patients with Kabuki syndrome type 2

Patient ID	1	2	3	4	5	6	7	8	9	10	11	12	Percentage n/N (%)
**Basic Information**													
Age (y)	7.3	4.3	12.3	0.3	5.3	10.7	6.8	7.0	4.5	35.0	34.0	32.0	
Sex	M	M	M	M	M	F	F	F	F	F	F	F	M:F = 5:7
Gestation (wk)	37 + 2	39	38 + 2	39 + 5	28	37 + 6	38	36 + 5	35	ND	ND	ND	
Birth length (cm)	50	50	50	50	38	49	50	48	48	ND	ND	ND	
Birth weight (kg)	2.75	3.1	3.5	3	1.25	2.85	3	2.9	2.5	ND	ND	ND	
Current height (cm)	113	100	152	62	90	135	122	117	94	160	148	158	
Height standard deviation score (SD)^a^	-2.48	-1.47	-0.23	-0.09	-5.85	-1.89	0.21	-1.12	-2.79	-0.11	-2.33	-0.48	
Current weight (kg)	17.5	22	44	5.5	10.6	38	23	18	13	55	40	55	
**Typical Clinical Features**													
Intellectual disability (HPO:0001249) Developmental delay (HPO:0001263)	+	+	+	/	+	-	-	+	+	-	+	-	7/12 (58.33)
IQ score^b^	38	42	46	/	40	72	78	65	66	92	68	80	
Typical dysmorphic features^c^	+	+	+	+	+	+	+	+	+	-	+	+	11/12 (91.67)
**Supportive Clinical Features**													
Constitutional													
Short stature (HPO:0004322)	+	-	-	-	+	-	-	/	+	-	+	-	4/11 (36.36)
Craniofacial													
Microcephaly (HPO:0000252)	+	-	-	+	+	-	-	-	+	-	+	-	5/12 (41.67)
Cleft palate (HPO:0000175)	-	-	-	-	-	-	+	+	-	-	-	-	2/12 (16.67)
Lip pits (HPO:0000196)	-	+	-	-	+	-	-	-	-	-	+	-	3/12 (25.00)
Oligodontia and/or abnormal incisors (HPO:0000677)	+	-	-	/	+	-	-	-	+	-	+	-	4/12 (33.33)
Progressive sensorineural hearing loss (HPO:0000408)	-	-	-	-	-	-	-	-	-	-	-	-	0/12 (0.00)
Cardiac													
Congenital heart defect (HPO:0002564)	+	+	+	-	+	-	-	-	+	-	-	-	6/12 (50.00)
Gastrointestinal													
Feeding difficulties (HPO:0011968)	+	+	-	+	+	+	+	+	-	ND	ND	ND	7/9 (77.78)
Genitourinary													
Malpositioned kidneys (HPO:0000086)	-	-	-	-	-	-	-	-	-	-	-	-	0/12 (0.00)
Hypospadias in male patients (HPO:0000047)	-	-	-	-	-	/	/	/	/	/	/	/	0/5 (0.00)
Musculoskeletal													
Hypotonia (HPO:0001252)	-	-	-	-	+	-	+	-	-	ND	ND	ND	2/9 (22.22)
Brachydactyly (HPO:0009381)	+	-	-	+	-	-	-	-	-	-	+	-	3/12 (25.00)
Non-traumatic joint dislocation (HPO:0001373)	-	-	-	-	-	-	-	-	-	-	-	-	0/12 (0.00)
Endocrinological													
Hyperinsulinemic hypoglycemia (HPO:0000825)	-	-	-	+	-	-	-	-	+	ND	ND	ND	2/9 (22.22)
Immunological													
Hypogammaglobulinemia (HPO:0004313)	-	-	-	-	-	-	-	-	-	-	-	-	0/12 (0.00)
Idiopathic thrombocytopenia purpura	-	-	-	-	-	-	-	-	-	-	-	-	0/12 (0.00)

*M* Male, *F* Female, *ND* No date, *HPO* Human phenotype ontology, *IQ* Intelligence quotient, *SD* Standard deviation

^a^The SD is based on the height and weight standardized growth charts for Chinese children and adolescents aged 0 to 18 years [10]

^b^Participants’ IQ scores were measured using the Wechsler Preschool and Primary Scale of Intelligence, Wechsler Intelligence Scale for Children–Fifth Edition, and Wechsler Adult Intelligence Scale–Fourth Edition

^c^Typical dysmorphic features include long palpebral fissures with eversion of the lateral third of the lower eyelid and two or more of the following: 1) arched and broad eyebrows with the lateral third displaying notching or sparseness; 2) short columella with a depressed nasal tip; 3) large, prominent, or cupped ears; and 4) persistent fingertip pad

**Table 2:** Sex-specific phenotypic difference in patients with Kabuki syndrome type 2

Variable	Males *N* = 5	Females *N* = 7	*P*-value
Age (mean ± SD, y)	5.88 ± 4.36	17.98 ± 13.62	0.087^a^
Height SD score (mean ± SD, SD)	-2.02 ± 2.35	-1.21 ± 1.15	0.876^a^
IQ score, median (P25–P75)	41 (38–42)	69 (65–72)	0.029^*b^
Intellectual disability (IQ < 70), n/N (%)	4/4 (100.00)	3/7 (42.86)	0.194^c^
Moderate-to-severe intellectual disability (IQ < 55), n/N (%)	4/4 (100.00)	0/7 (0.00)	0.003^**c^
Typical dysmorphic features, n/N (%)	5/5 (100.00)	6/7 (85.71)	1.000^c^
Short stature, n/N (%)	2/5 (40.00)	2/7 (28.57)	1.000^c^
Microcephaly, n/N (%)	3/5 (20.00)	2/7 (0.00)	0.558^c^
Cleft palate, n/N (%)	0/5 (0.00)	2/7 (28.57)	0.470^c^
Lip pits, n/N (%)	2/5 (40.00)	1/7 (14.29)	0.523^c^
Oligodontia, n/N (%)	2/4 (50.00)	2/7 (28.57)	0.576^c^
Congenital heart defect, n/N (%)	5/5 (100.00)	1/7 (14.29)	0.015^*c^
Feeding difficulties, n/N (%)	4/5 (80.00)	3/4 (75.00)	1.000^c^
Hypotonia, n/N (%)	1/5 (20.00)	1/5 (20.00)	1.000^c^
Brachydactyly, n/N (%)	2/5 (40.00)	1/7 (14.29)	0.523^c^
Hyperinsulinemic hypoglycemia, n/N (%)	1/5 (20.00)	1/5 (20.00)	1.000^c^

*IQ* Intelligence quotient, *SD* Standard deviation, *P25* 25th percentile, *P75* 75th percentile

Significant at *P < 0.05, ** *P* < 0.01, and *** *P* < 0.001

^a^*P*-value according to the t test

^b^*P*-value according to the rank sum test

^c^*P*-value according to the Fisher exact test

Regarding genotype, nine different variant sites were found in these 12 patients, among which Patients 10, Patient 11, and Patient 12 were the mothers of Patient 2, Patient 4, and Patient 5, respectively. Other patients were confirmed to be de novo. Among these variants, six were null variants (four truncating, one nonsense, and one splicing), and three were missense variants. The variant of Patient 5 and Patient 12 can only be evaluated as “Uncertain significance,” but they were included in this study because of their typical clinical manifestations. Other mutations can be classified as “Pathogenic” or “Likely Pathogenic” variants according to the American College of Medical Genetics and Genomics (ACMG) guideline (Table 3).

**Table 3:** Variation categories of these 12 patients with Kabuki syndrome type 2

Patient	*KDM6A* variant	Protein	Heredity	ACMG
1	c.404G > A	p. Gly135Asp	De novo	Likely pathogenic
2	c.748 + 2 T > C	/	Mother	Pathogenic
3	c.2516-2519del	p. Asn839Metfs*27	De novo	Pathogenic
4	c.288-305del	p. Phe96fs	Mother	Likely pathogenic
5	c.871G > A	p. Gly291Arg	Mother	Uncertain significance
6	c.1834C > T	p. Arg612*	De novo	Pathogenic
7	c3217-3218G	p.1073R > Rfs*8	De novo	Pathogenic
8	c.3206A > G	p. Asp1069Gly	De novo	Likely pathogenic
9	c.1909-1912delTCTA	p. Ser637ThrfsTer53	De novo	Pathogenic
10	c.748 + 2 T > C	/	Unknown	Pathogenic
11	c.288-305del	p. Phe96fs	Unknown	Likely pathogenic
12	c.871G > A	p. Gly291Arg	Unknown	Uncertain significance

### Relationship between ID and the sexes

**Fig. 1:**
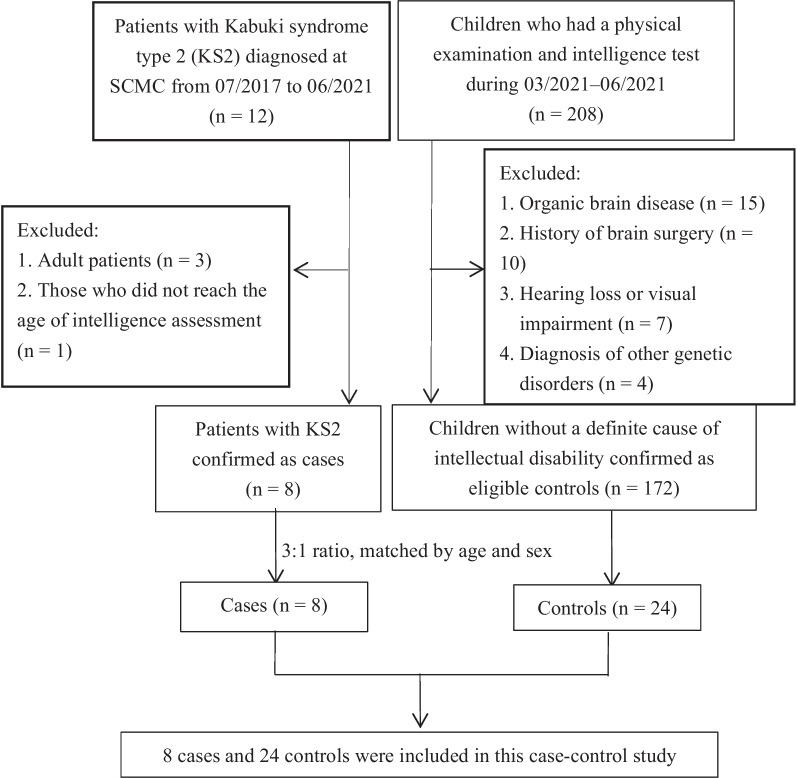
Flow chart of this matched case–control study

For further analysis of this phenotype, we stratified the case and control groups by sex, and the results changed. In the case group, except for three adults, one patient who was younger than four years of age was not able to perform the intelligence test, so we finally matched eight patients in the case group with 24 children in the control group (Fig. 1). Although the IQ value of female patients was still lower than that of the controls, there was no significant difference in the incidences of ID or moderate-to-severe ID between the case and control groups (*P* > 0.05). The same comparison for male patients had the opposite result, that is, the IQ score of male patients was significantly lower than that of the controls (*P* < 0.001), and the incidences of various severities of ID were significantly higher in male patients than in the controls (*P* < 0.05) (Table 4). Likewise, the IQ values between the male patient group and female patient group were significantly different, as seen in Table 2 (*P* = 0.029).

**Table 4:** Risk of intellectual disability between patients with Kabuki syndrome type 2 and age-sex-matched controls

	**KS2 cases** ***N* = 8**	**Controls** ***N* = 24**	***P*-value**
**Age (y)**, median (P25–P75)	6.87 (4.50–7.25)	6.92 (4.83–7.42)	1.000^b^
**IQ score**, median (P25–P75)			
Total	53.50 (40–66)	88.00 (73–108)	< 0.001^***b^
Males	41 (38–42)	99 (76–117)	< 0.001^***b^
Females	69 (65–72)	96 (77–105)	0.042^*b^
**Intellectual disability** (IQ < 70), n/N (%)			
Total	6/8 (75.00)	4/24 (16.67)	0.005^**c^
Males	4/4 (100.00)	2/12 (16.67)	0.008^**c^
Females	2/4 (50.00)	2/12 (16.67)	0.245^c^
**Moderate-to-severe intellectual disability** (IQ < 55), n/N (%)			
Total	4/8 (50.00)	2/24 (8.33)	0.023^*c^
Males	4/4 (100.00)	2/12 (16.67)	< 0.001^***c^
Females	0/4 (0.00)	1/12 (8.33)	1.000^c^

*KS2* Kabuki syndrome type 2, *IQ* Intelligence quotient, *SD* Standard deviation, *P25* 25th percentile, *P75* 75th percentile

Significant at ^*^P < 0.05, ^**^P < 0.01, and ^***^P < 0.001

^a^*P*-value according to the t test

^b^*P*-value according to the rank sum test

^c^*P*-value according to the Fisher’s exact test

## Discussion

In our study, we definitively diagnosed 12 patients with KS2 based on clinical symptoms and genetic testing and compared the differences in the incidence of various phenotypes between male and female patients with KS2 using a matched case–control study design. A typical dysmorphic feature is an emblematic clinical manifestation of KS. In previous studies, we found that almost all Chinese KS patients demonstrated the characteristic facial features of KS [11]. Makrythanasis et al. established a score list in which half of the scores were derived from facial features. Their study indicated that the score of the KMT2D variant group was higher than that of the non-KMT2D group [6], which was confirmed in our previously reported cases (KS1 4.72 vs KS2 3.33) [11]. In this cohort, Patient 10 (mother of Patient 2) showed eyebrows with lateral sparseness but lacked the other facial features, suggesting that female patients with KS2 may have a lower score than those of the non-KMT2D group. Whether the scoring system mentioned above is applicable to female patients with the *KDM6A* variant needs further exploration. It may not be appropriate to evaluate female patients with KS2 and male patients with KS2 when exploring the phenotype of KS2. Furthermore, in our study, we diagnosed three adult patients with KS2 coincidentally during parental verification due to the birth of KS2 boys. Recent studies suggest that the diagnosis of KS in adulthood is challenging. Their facial deformities are less obvious in adult patient populations, and even the most typical lower eyelid ectropion may disappear [12]. This may explain the lack of Typical dysmorphic features in Patient 10. This suggests that facial scoring may not be applicable to adult KS patients either.

Our statistical findings showed significant differences in the IQ scores, incidence of ID, and CHD between female and male patients with KS2.For ID, there is currently no effective treatment, but this is one of the most confusing parts for families of patients with KS. Our study evaluated the IQ scores of patients with KS2 using the Wechsler Intelligence Test and obtained IQ scores of adult patients with KS2. Cristina et al. tested the IQ of KS1 patients and found an IQ score of 67 ± 24.9 (*n* = 6), suggesting that a small number of patients with KS have severe ID [13]. When analyzing patients with KS2, we found that the median IQ score of female patients in our case group reached 69 points, which was the highest score for mild ID, while male patients had an IQ score of only 41 points, almost reaching the diagnostic criteria for severe ID. In our study, there was a significant statistical difference in the incidence of moderate-to-severe ID (IQ < 55) between male and female patients. Additionally, two of the four male patients’ IQ scores were less than 40, and the rate of severe ID reached 50%. Although the ID score of KS described in the past indicates mostly mild-to-moderate ID [14], in our study, severe ID was a common finding in male patients with KS2. Previous studies have suggested a high frequency of psychiatric comorbidities, including anxiety and depression, in patients with KS, which requires further evaluation in our cohort in the future.

The incidence of CHD in patients with KS varied in previous reports from approximately 28% to 80% [15–17]. Maria et al. summarized the pattern of CHDs in 28 KS patients with KS (27 KS1, 1 KS2); 19 of them had CHD with coarctation of the aorta, aortic valve, atrial septal defect, and ventricular septal defect [15]. In our study, we completed echocardiography of 12 patients with KS2, five of whom had CHD. Patients 1, 2, 3, and 9 had an atrial septal defect (ASD), and Patient 4 had pulmonary stenosis. Previously published studies have suggested that ASD is the most common type of CHD in patients with variants of *KDM6A*, which is consistent with our findings [3, 15, 18]. Additionally, we further found that the incidence of CHD in male patients with KS2 was significantly higher than that in female patients with KS2. Thus, we assume that CHD also manifests as a sex-specific difference.

In our study, clinical manifestations including short stature, microcephaly, cleft palate, feeding difficulties, hypotonia, etc., had no difference in the incidence between KS2 male and KS2 female patients. But this may be related to the small number of cases being studied.

As mentioned earlier, KS1 patients tend to have more typical clinical manifestations than KS2 patients. However, on the contrary, it has been reported that the incidence of hypoglycemia was higher in patients with KS2 than in those with KS1. Kai et al. reported ten cases of hyperinsulinemic hypoglycemia among patients with KS, among which five cases were KS2 [19]. In our cohort, two patients were diagnosed with hyperinsulinemic hypoglycemia, among whom Patient 4 required maintenance with glucose transfusion treatment due to persistent hypoglycemia and severe feeding difficulties. When his intravenous glucose level was 2.0 mmol/l, his insulin level was up to 5.5 µIU/ml, and his C-peptide level was 0.46 nmol/l, which indicated hyperinsulinemic hypoglycemia. After obtaining informed consent from the patient’s parents, we administered diazoxide for treatment. During the treatment, the peripheral blood glucose level was maintained at about 4 mmol/L, and there were no adverse reactions. Although the etiology of hyperinsulinemic hypoglycemia in patients with KS is still unclear, its reported onset age in these patients is young, suggesting that KS may be one of the potential genetic factors of hypoglycemia in infants and young children [19–21]. The mechanism of pathogenic variation in the *KDM6A* gene leading to hyperinsulinemic hypoglycemia is unclear.

**Fig. 2:**
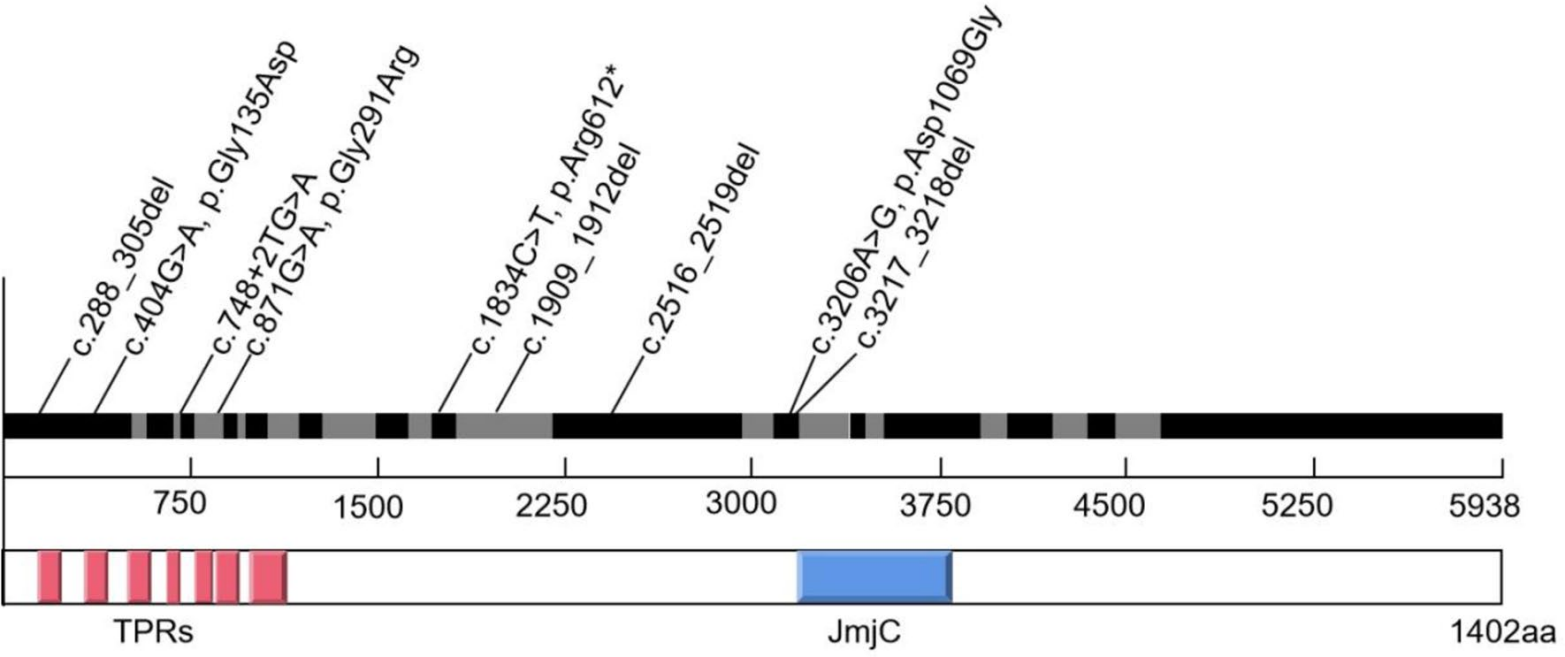
*KDM6A* genotype of 12 patients with Kabuki syndrome type 2

The *KDM6A* gene consists of 29 exons and contains a key domain, Jumonji C (JmjC), whose integrity is essential for maintaining histone demethylase activity. The loss of function of the *KDM6A* gene causes KS2, and we found nine different variants in 12 patients with KS2. Four of them were frameshift variants located in exons 1, 16, 17, and 19, and one was a nonsense variant located in exon 15. The three missense variants were located in exons 1, 6, and 19 (Fig. 2). Combining our data with previously published cases showed no obvious hot spots or regions [5, 22]. However, all null variant sites in our cases were located in front of the JmjC domain, causing premature termination of gene transcription.

Histone modification is an important part of epigenetics, and different histone modifications have different effects on downstream genes. Methylation of H3K4 plays a role in transcriptional activation, whereas methylation of H3K27 plays a role in transcriptional inhibition [23]. The methylation markers of H3K27 were labeled with Polycomb proteins and removed by KDM6 demethylases, of which *KDM6A* is an important member [24, 25]. *KDM6A* is located on the X chromosome, not in the pseudoautosomal region segment, but escapes X chromosome inactivation [26]. Although UTX homologue UTY is found on the male Y chromosome, it was thought to lack demethylation in vitro. As for KS patients with the same variant site, the difference in phenotype between female and male patients may result from the functional difference between UTX and UTY. However, some recent studies have shown that UTY still has an important physiological role [27]. Female homozygous mice could not survive, while hemizygous males could reflect the residual function of UTY [28]. Human *KDM6A* and UTY transcripts show up to 88% complementary DNA homology and 86% predicted amino acid homology [29]. Both carry the JmjC domain, which is extremely important for demethylation [30]. However, based on our case–control studies and previously reported cases, there are differences in the phenotypic severity of KS2 between the sexes. This phenomenon still requires more case support, and the mechanism still needs to be further explored.

### Limitations

Although our study described a KS2 cohort with 12 patients, we also conducted a case–control study that revealed the phenotypic differences between male and female patients with KS. Nevertheless, some limitations should be noted. First, the number of cases in this study was limited. These 12 patients with KS2 were diagnosed within four years at a single center. Considering the rarity of KS2, the diagnosis rate was not low. In future studies, we will collect more cases and conduct multi-center studies to further analyze the phenotype and genotype of patients with KS2. Second, most of the children in our control group did not undergo genetic testing, because genetic testing is not a routine test item for normal children. Therefore, we could only exclude children with other causes of ID based on imaging or laboratory examinations. Third, we did not dynamically monitor the level of patients’ intellectual development and did not repeatedly evaluate patients’ IQ, and this is what we need to further improve in the future. By doing this, more convincing results can be obtained. In addition, for the assessment of CHD, the data we obtained may be biased, because whether the three adult patients had CHD in childhood could only be recalled without objective evidence. In future studies, a greater collection of children’s cases can solve this problem.

## Conclusions

This study analyzed and summarized the spectrum of genotype and phenotype of 12 KS2 patients. In addition, through a case–control study, we further confirmed that there were sex-specific differences in the clinical phenotypes of patients with KS2. Compared to female patients, male patients had a higher incidence of CHD and significant mental retardation. The phenotypes of female patients were always milder.

## Data Availability

No datasets were generated or analysed during the current study.

## Abbreviations

KS: Kabuki syndrome
KS1: KS type
KS2: Kabuki syndrome type 2
ACMG: American College of Medical Genetics and Genomics
IQ: Intelligence Quotient; ID: intellectual disability
CHD: Congenital heart disease
SCMC: Shanghai Children’s Medical Center
ASD: Atrial septal defect
